# Comparative Hypothalamic Proteomic Analysis Between Diet-Induced Obesity and Diet-Resistant Rats

**DOI:** 10.3390/ijms26052296

**Published:** 2025-03-05

**Authors:** Pengjiao Xi, Shuhui Ma, Derun Tian, Yanna Shen

**Affiliations:** 1 College of Medical Technology, Tianjin Medical University, Tianjin 300203, China; xipj@tmu.edu.cn (P.X.);; 2 Department of Human Anatomy and Histology, Tianjin Medical University, Tianjin 300070, China

**Keywords:** proteomic, obesity, diet-induced obesity resistance, hypothalamus

## Abstract

Obesity arises from a complex interplay of genetic and environmental factors. Even among individuals with the same genetic predisposition, diet-induced obesity (DIO) exhibits varying degrees of susceptibility, which are categorized as DIO and diet-induced obesity resistance (DR). The hypothalamus plays a pivotal role in regulating energy homeostasis. This study performed a comparative hypothalamic proteomic analysis in DIO and DR rats to identify differentially expressed proteins (DEPs) associated with alterations in body weight. Male Sprague Dawley rats were fed either a standard chow diet or a high-fat diet for 12 weeks. DIO rats exhibited the most rapid weight gain compared to both the control and DR rats. Despite consuming similar caloric intake, DR rats exhibited less weight gain relative to DIO rats. Proteomic analysis revealed 31 DEPs in the hypothalamus of DR rats compared to DIO rats (with a false discovery rate (FDR) < 1%). Notably, 14 proteins were upregulated and 17 proteins were downregulated in DR rats. Gene ontology analysis revealed an enrichment of ion-binding proteins, such as those binding to Fe^2+^, Zn^2+^, Ca^2+^, and Se, as well as proteins involved in neuronal activity and function, potentially enhancing neuronal development and cognition in DR rats. The DEPs pathway analysis via the Kyoto Encyclopedia of Genes and Genomes (KEGG) implicated starch and sucrose metabolism, antigen processing and presentation, and the regulation of inflammatory mediator affecting TRP channels. Western blotting confirmed the proteomic findings for TRPV4, CaMKV, RSBN1, and BASP1, which were consistent with those obtained from Tandem Mass tag (TMT) proteomic analysis. In conclusion, our study highlights the hypothalamic proteome as a critical determinant in the susceptibility to DIO and provides novel targets for obesity prevention and treatment.

## 1. Introduction

Obesity is a major risk factor for cardiovascular disease, type 2 diabetes, cancer, and other diseases. With the improvement of living standards and changes in lifestyle, the prevalence of obesity has increased year by year, becoming a critical global public health issue [1]. It is reported that there are currently nearly 1.9 billion overweight individuals worldwide, including over 650 million classified as obese. Each year, approximately 2.8 million deaths are attributed to obesity and obesity-related diseases [2]. Therefore, identifying effective therapeutic targets for obesity has become an urgent priority.

Apart from hereditary predispositions, a considerable number of obesity cases can be correlated with the chronic intake of hypercaloric diets, leading to an imbalance in energy homeostasis and subsequent adiposity accumulation. However, a small group of individuals, known as obesity-resistant, remain lean despite consuming high-calorie diets [3,4]. Similarly, in rodent models, individual differences in sensitivity to obesity are observed. For example, after being fed a high-fat diet (HFD) for four months, more than 50% of Sprague–Dawley (SD) rats will develop obesity, known as diet-induced obesity (DIO). Conversely, approximately 20% of the rats remain resistant to obesity, designated as diet-induced obesity resistance (DR) [5,6]. Previous research has shown that DR rats exhibit increased uncoupling protein 1(UCP1) protein expression in brown adipose tissue (BAT), which enhances thermogenesis and promotes weight maintenance [4,7]. Other studies suggest that DR mice have superior fat oxidation capabilities compared to their DIO counterparts [8]. Additionally, Piché et al. reported that gut microbiota composition and their metabolites play an important role in the development of DR [9]. Despite these findings, the precise mechanism underlying obesity resistance remains unclear.

The imbalance of energy metabolism is the key driver of obesity. The hypothalamus, a central brain region, plays a crucial role in regulating energy homeostasis and food intake [10]. Within the hypothalamus, specialized neurons monitor peripheral metabolic status to maintain the balance between food consumption and energy metabolism. Disruption in the development of key hypothalamic nuclei can impair this balance between energy intake and expenditure, leading to rapid and excessive weight gain. Cornier et al. demonstrated that DR individuals exhibit greater sensitivity to short-term energy fluctuations [11]. For example, neuronal responses to food intake vary significantly under conditions of undereating and overeating. Similarly, Souza proposed that insufficient activity of hypothalamic neurons contributes to obesity development [12]. Additionally, the abnormal expression of dysregulated proteins in the hypothalamus is also an important cause of obesity. For instance, downregulation of hypothalamic TGR5 exacerbates obesity by reducing sympathetic nerve activity [13]. Therefore, identifying differentially expressed proteins (DEPs) in the hypothalamus of DIO and DR rats will provide new targets for the prevention and treatment of obesity.

In this study, we performed a comparative proteomic analysis of the hypothalamus in DR and DIO rats using the Tandem Mass tag (TMT) methodology. Our objective was to identify DEPs between the two groups and explore the underlying mechanisms contributing to obesity.

## 2. Results

### 2.1. The Differences in Metabolic Characteristics Between DIO and DR Rats

**Figure 1:**
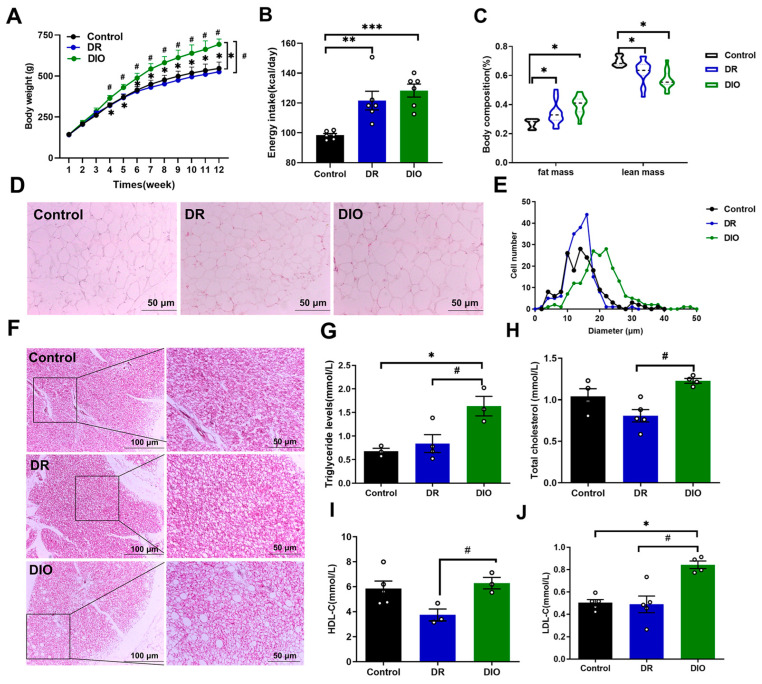
Metabolic characteristics of DR and DIO rats were assessed. (**A**) Body weight was recorded weekly until week 12 (n = 10/group). (**B**) Average daily energy intake (kcal/day). (**C**) Fat mass and lean mass. (**D**) Representative images of WAT stain with H&E (magnification: 200×, scale bar: 50 μm), (n = 6/group). (**E**) Quantification of adipocyte size of WAT described in D. (**F**) H&E staining of BAT (magnification: 100×, scale bar: 100 μm; magnification: 200×, scale bar: 50 μm), (n = 6/group). (**G**–**J**) The concentrations of triglycerides (**G**), total cholesterol (**H**), HDL-C (**I**), and LDL-C (**J**) were determined, (n = 3–5/group). Data were presented as mean ± SEM. * *p* < 0.05, ** *p* < 0.01 and *** *p* < 0.001 vs. control group; # *p* < 0.05 DR vs. DIO group.

Compared with rats who are fed with a standard chow diet (Control), HFD feeding significantly increased body weight in DIO rats. In contrast, HFD did not induce weight gain in DR rats, with DR rats remaining the lightest among all groups (Figure 1A). After calculating the caloric content in the diets, both DIO and DR rats showed higher caloric intake than the control group (Figure 1B). HFD feeding markedly increased fat mass and decreased lean mass in DIO and DR rats compared to control rats (Figure 1C). In the white adipose tissue (WAT), HFD notably increased adipocyte volume. However, compared to the DIO group, DR rats exhibited smaller white adipocyte volume and a higher frequency of small-volume adipocytes (10~20 μm) (Figure 1D,E). The H&E staining revealed an increased number of lipid droplets in the BAT of DIO rats compared to DR rats, suggesting an alteration in adipocyte composition (Figure 1F). Additionally, DR rats had lower levels of triglyceride, total cholesterol, high-density lipoprotein cholesterol (HDL-C), and low-density lipoprotein cholesterol (LDL-C) compared to control and DIO rats (Figure 1G–J). These results suggest that DR rats are able to maintain normal weight despite being exposed to the same HFD conditions, indicating that their energy balance regulation system has strong compensatory capacity.

### 2.2. Proteomics Analysis of Hypothalamus in DIO and DR Rats

**Figure 2:**
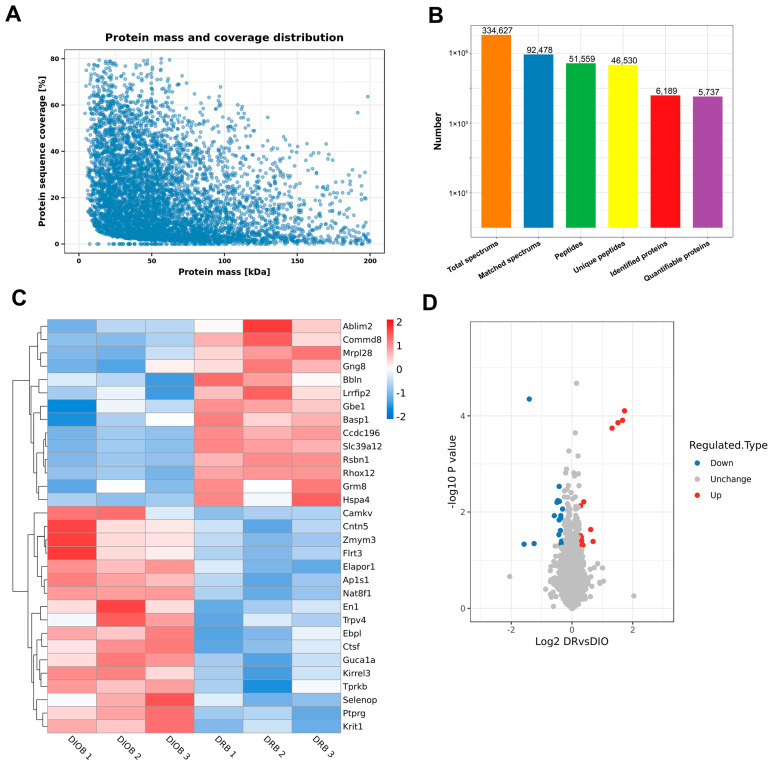
Differentially expressed proteins (DEPs) in DR vs. DIO group. (**A**) Distribution of protein mass and coverage. (**B**) Statistical analysis of mass spectrometry data results. (**C**) A heatmap of DEPs in the hypothalamus of DR and DIO rats. Protein expression changes were fold change > 1.2 or <1.2 and *p* < 0.05. Upregulated and downregulated proteins are represented as red and blue dots, respectively. Proteins that were not differentially expressed are presented as gray dots. (**D**) Volcano diagrams of DEPs. The abscissa represented the difference in proteins folds (Log 2), the vertical axis represented *p* value (−log10).

The hypothalamus, a critical brain region for energy balance regulation, was examined for DEPs. After trypsin digestion and HPLC/MS analysis, we identified 51,559 distinct peptides and 6189 proteins with a false discovery rate (FDR) < 0.01 (Figure 2A,B). Comparative analysis between DR and DIO rats revealed 31 DEPs (14 upregulated and 17 downregulated in DR rats), with significant changes defined by FDR < 0.01 and log2 fold change > 1.2 (Table 1 and Table 2). A heatmap illustrating protein expression patterns is shown in Figure 2C, while a volcano plot of the DEPs is presented in Figure 2D. Interestingly, analysis of datasets revealed that the DEPs in the hypothalamus of DR rats were primarily classified into two categories: ion-binding proteins (e.g., Fe^2+^, Zn^2+^, Ca^2+^, Se-binding proteins) and proteins associated with neuronal activity and function. For instance, calcium-related proteins included brain acid soluble protein 1(BASP1), CaM kinase-like vesicle-associated protein (CaMKV), transient receptor potential cation channel subfamily V member 4 (TRPV4), and guanylate cyclase activator 1A (GUCA1A). Zinc-binding proteins included solute carrier family 39 member 12(SLC39A12), actin-binding LIM protein 2(ABLIM2) and Zinc finger MYM-type-containing 3 (ZMYM3). These results suggest that, unlike DIO rats, multiple ion channels may be altered in DR rats. Additionally, the differentially abundant proteins were associated with axon-genesis (BASP1, CNTN5, and FLRT3), neuron development (GNG8, SLC39A12), and neuronal project (KIRREL3, TRPV4 and PTPRG). Previous studies have found that zinc ions in the brain are positively correlated with cognitive level [14]. Excessive deposition of calcium ions may also lead to neuronal damage. These results suggest that the neural development and cognitive levels in the hypothalamus of DR rats may differ from those of DIO rats.

### 2.3. GO Analysis of DEPs

**Figure 3:**
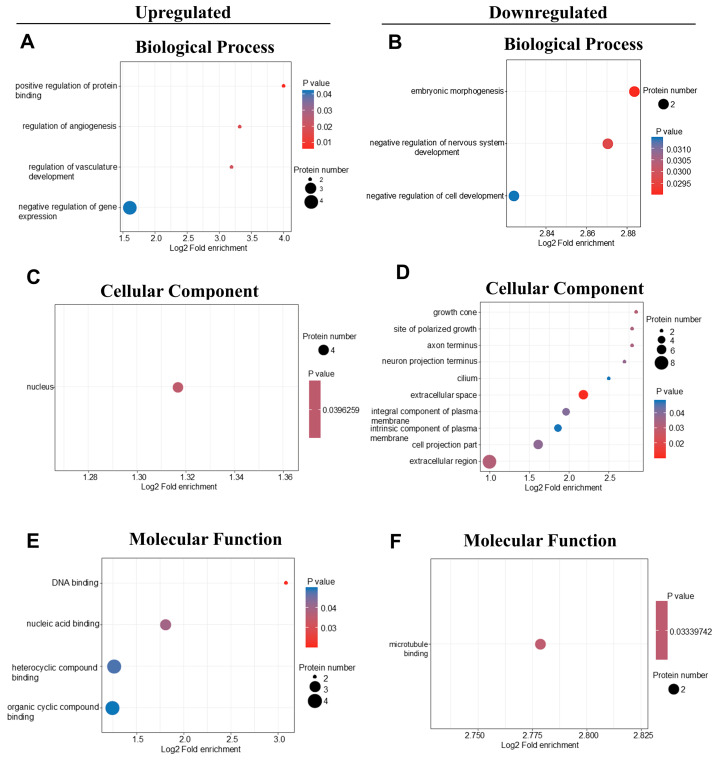
GO enrichment analysis in DR vs. DIO group. The graph showed enrichment results in the three categories: biological process, cellular component and molecular function. (**A**,**C**,**E**) Upregulated proteins in DR vs. DIO rats were conducted. (**B**,**D**,**F**) Downregulated proteins were analyzed by GO enrichment.

Gene ontology (GO) analysis provided insights into the biological functions of DEPs across three categories: biological process, cellular component, and molecular function. For the 14 upregulated proteins in DR vs. DIO comparison, GO annotation of biological processes indicated significant enrichment in the positive regulation of protein binding and the regulation of angiogenesis (Figure 3A). In contrast, for the downregulated proteins, the two most enriched biological processes were embryonic morphogenesis and the negative regulation of nervous system development (Figure 3B). Notably, the proteins TRPV4, CaMKV, PTPRG and CNTN5 proteins were significantly downregulated in these processes (Table 2).

In the cellular component category, enriched terms for downregulated proteins included growth cone, axon terminus, and neuron projection terminus, emphasizing the impact of obesity on neuronal function (Figure 3D). However, the upregulated proteins mainly affect the cell nucleus (Figure 3C). Therefore, in terms of molecular function, the upregulated proteins are mainly involved in DNA binding, while the downregulated proteins mainly act on the binding of neural microtubules (Figure 3E,F).

### 2.4. KEGG Pathway Enrichment Analysis of DEPs

**Figure 4:**
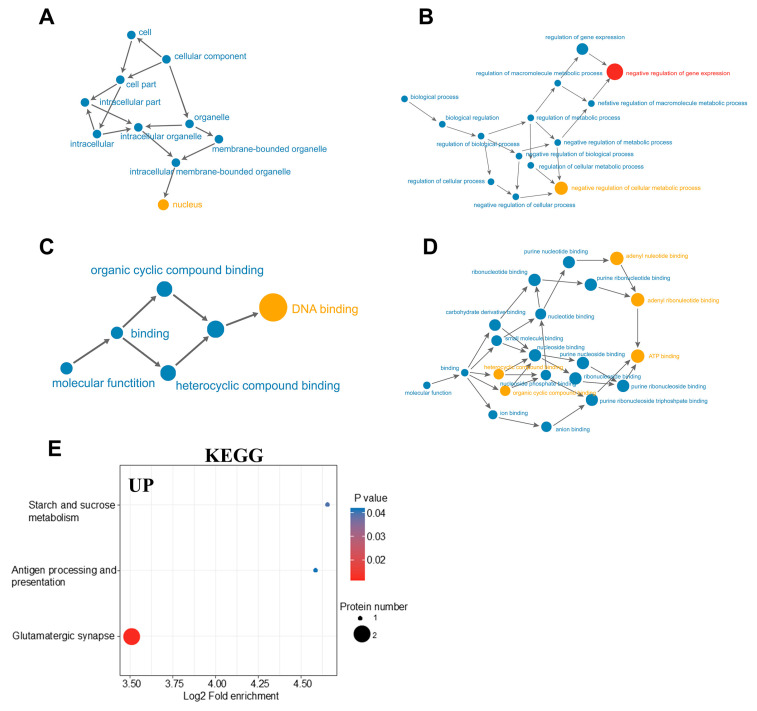
(**A**–**C**) Directed acyclic map of GO enrichment of upregulated proteins in hypothalamus of DR rats. (**D**) Directed acyclic map of GO enrichment of downregulated proteins in hypothalamus of DR rats. (**E**) Enrichment of upregulated proteins in KEGG pathway.

Figure 4A–D showed the GO enrichment analysis results of DEPs using directed acyclic graph. The circles in the figure represented the GO classification in which the differential proteins are located, with red representing extremely significant DEPs (*p* value ≤ 0.01), yellow representing significant differential proteins (*p* value ≤ 0.05), and blue representing non-significant enrichment. The lines with arrows represented the upper and lower levels of the GO classification, and the circle size denoted the degree of enrichment. Figure 4A–C showed the network connections of cellular components, biological processes, and molecular functions involved in upregulated proteins. The results showed that the network connections involved in upregulating DEPs were relatively simple, and the relationship between upstream molecules and downstream molecules was clear. On the contrary, the network connections involved in downregulating the molecular functions of proteins were complex (Figure 4D).

KEGG pathway analysis revealed that upregulated proteins in the DR groups were associated with starch and sucrose metabolism, antigen processing and presentation, as well as glutamatergic synapse (Figure 4E). The downregulated proteins predominantly included those involved in the regulation of TRP channels by inflammatory mediators and cellular senescence [15]. Chronic consumption of a HFD disrupts glucose metabolism and triggers persistent low-grade inflammation. These findings underscore a distinctive protein expression profile in the hypothalamus of DR rats, which may account for their resilience to obesity.

### 2.5. Analysis of Upregulated Proteins in Hypothalamus of DIO and DR Rats

**Figure 5:**
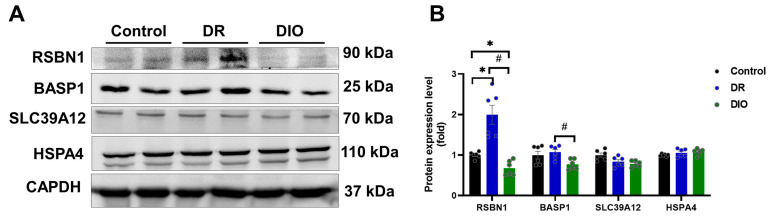
(**A**) The expression levels of upregulated proteins were assessed using Western blotting, (n = 6/group). (**B**) Densitometric analysis quantified the expression levels of proteins. * *p* < 0.05 vs. Control group; ^#^ *p* < 0.05 DR vs. DIO group.

Focusing on DEPs with fold changes > 1.5 times and *p* < 0.05 (FDR < 0.01), six upregulated proteins were identified in the DR group: RHOX12, BASP1, CCDC196, SLC39A12, HSPA4, and RSBN1(Table 3). Western blotting confirmed increased expression of RSBN1 and BASP1 in the DR group, consistent with the proteomic data (Figure 5A,B). Antibodies for RHOX12 and CCDC196 were unavailable, while SLC39A12 and HSPA4 showed no significant differences.

### 2.6. Analysis of Downregulated Proteins in Hypothalamus of DIO and DR Rats

**Figure 6:**
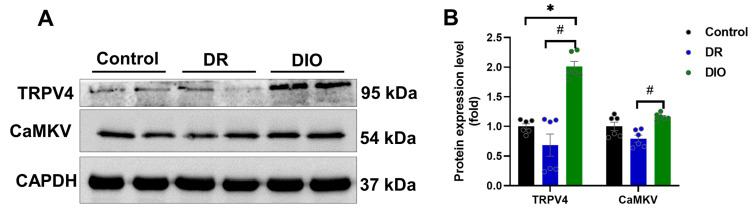
(**A**) The expression levels of downregulated proteins TRPV4 and CaMKV were assessed by Western blotting, (n = 6/group). (**B**) Densitometric analysis quantified the expression levels of TRPV4 and CaMKV proteins. * *p* < 0.05 vs. Control group; ^#^ *p* < 0.05 DR vs. DIO group.

Two proteins, TRPV4 and CaMKV, were significantly downregulated in the DR group [15]. Western blotting validated their reduced expression (Figure 6A,B). TRPV4, a calcium-permeable ion channel, and CaMKV, a pseudokinase in the Ca^2+^/calmodulin-dependent kinase family, play critical roles in neuronal function, axon growth, and synaptic plasticity. The abnormal expression of these proteins suggests a role in obesity-related neuronal dysfunction and may serve as potential therapeutic targets.

## 3. Discussion

In this study, we identified changes in the hypothalamic proteome of DR and DIO rats. While proteomic changes in obesity models have been previously investigated, the comparison of the hypothalamus proteomes between the DR and DIO groups using the TMT approach represents a novel contribution. Moreover, a comprehensive analysis of the hypo-thalamic global proteome uncovered a range of proteins that have not been previously reported in studies on obesity or energy metabolism. For instance, cation binding proteins, such as CaMKV and TRPV4, were identified as particularly abundant and pivotal in the central nervous system (CNS). The dysregulation of these proteins appears to play a crucial role in the function of hypothalamic neurons, axon growth, and other processes.

It is widely recognized that, in addition to genetic factors, the two primary contributors to obesity are excessive energy intake and reduced energy expenditure. This study reveals that, under the same genetic background, DIO rats exhibited a more rapid increase in body weight starting from 4 weeks compared to DR rats, with this difference being statistically significant. Upon measuring daily food intake and converting it into consumption energy, no difference in energy intake was observed between DIO and DR rats. However, the body weight of DR rats was significantly lower than that of DIO rats and even comparable to the control rats. The size of white adipocytes in DR rats was similar to that of control rats, but significantly smaller than those in DIO rats. Additionally, BAT is a thermogenic organ that contributes to non-shivering thermogenesis [16]. Long-term HFD can cause brown adipocytes to become white. However, the brown adipocytes of the DR rats were similar to those of the control rats, with no significant accumulation of fat droplets. In addition, we also measured the concentrations of leptin and thyroid hormones in the serum (Figure S1A,B). We found that, compared with the control and DR rats, the concentration of leptin in the serum of DIO rats was significantly increased. The serum leptin content of DR rats was the lowest. Leptin is a hormone secreted by adipose tissue that can inhibit food intake. Research has confirmed that leptin levels are strongly related to body fat levels. Our results are also consistent with previous reports [17]. An increase in thyroid hormones can enhance the body’s basal metabolic rate. Research has shown that patients with significant hypothyroidism typically experience weight gain, while those with hyperthyroidism often experience weight loss [18]. In this study, there was no change in thyroid hormone levels in DIO and DR rats, indicating that weight loss in DR rats is not attributed to thyroid dysfunction. These results suggest that DR rats have a strong compensatory ability for long-term HFD.

The hypothalamus is an important part of the balance system that regulates appetite and energy balance. Located near the median eminence, this is an area rich in capillaries that can be utilized by circulating hormones, nutrients, and metabolites. The hypothalamus acts as an ideal relay center to transmit circulating peripheral signals to the brain [19]. Proteomic analysis revealed 31 DEPs in the hypothalamus of DR rats compared to DIO rats (with FDR < 1%). Notably, 14 proteins were upregulated and 17 proteins were downregulated in DR rats. Western blotting confirmed the proteomic findings of TRPV4 and CaMKV, which are highly expressed in the hypothalamus of DR rats, consistent with the results of TMT proteomic analysis. TRPV4, a member of the transient receptor potential (TRP) channel family, functions as a calcium-permeable ion channel protein and is widely expressed in neurons and glial cells [20]. Studies have underscored the multifaceted role of TRPV4 in maintaining adipose tissue stability, promoting BAT production, and regulating macrophage function and energy metabolism [21,22]. Kumar et al. have reported that TRPV4 promotes macrophage activity in lung inflammation [23], while Ye Li et al. have identified TRPV4 as a key regulator of fat oxidation metabolism, inflammation, and energy balance [24]. Notably, TRPV4 knockout mice exhibited elevated thermogenic gene expression in adipose tissue, attenuated HFD-induced obesity, and decreased levels of inflammatory cytokines such as TNF-α and IL-6. However, conflicting evidence suggest that mice lacking TRPV4 genes are more susceptible to obesity, which underscores the debate regarding its role in obesity regulation [25]. In our previous study, we demonstrated that hypothalamic TRPV4 overexpression increased obesity susceptibility [15]. Conversely, adipose tissue-specific TRPV4 knockout mice exhibited obesity resistance by promoting the browning of white adipocytes [26]. These findings imply that the impact of TRPV4 on obesity may be tissue-dependent, warranting further investigation. Collectively, these findings suggest that dysregulated TRPV4 expression could be a promising therapeutic target for the prevention and treatment of obesity.

CaMKV is a calmodulin-binding protein that plays an important role in dendritic spine activity, synaptic plasticity, and hippocampal memory function. Studies have confirmed that dendritic protein synthesis is a key mechanism underlying the development and plasticity of neural circuits [27]. CaMKV modulates the density of dendritic spines through Ca^2+^/calmodulin signaling pathway. In the present study, we observed a significant downregulation of proteins involved in the negative regulation of nervous system development in the hypothalamus of DR rats, including CaMKV. Conversely, CaMKV protein expression was significantly increased in DIO rats. Several studies have shown that obesity and diabetes can impair hippocampal plasticity, potentially leading to cognitive decline, emotional disturbances, and overeating [28,29]. The decrease in CaMKV expression that we observed may alleviate the adverse effect of HFD on neural plasticity, which needs further verification.

GO enrichment analysis identified “binding” as the main molecular function. Notably, the DEPs associated with protein binding are involved in the binding of various cations, including calcium (Ca^2+^), zinc (Zn^2+^), and iron (Fe^2+^). Ca^2+^-related proteins include CaMKV, TRPV4, BASP1, and GUCA1A. Calcium has been identified as a pivotal mediator in the communication between neuronal synapses and the nucleus, governing synaptic activity. Decreased intracellular Ca^2+^ levels have been shown to be neurotoxic and pathogenic factors driving the advancement of neurodegenerative diseases, as well as to the augmentation of neuronal autophagy [29]. Zinc (Zn^2+^), the second most abundant essential trace element in humans, plays an important role in various physiological processes, such as cell proliferation, apoptosis, transcription, growth, and immunity. Studies have shown that individuals with obesity often exhibit reduced serum zinc levels, and zinc supplementation has been shown to improve weight and insulin resistance in the obese [7,30]. De Oliveira et al. found that zinc supplementation in rats reduced obesity-related neuroinflammation and enhanced metabolic function and memory in rats [30]. Recent studies suggest that the dysfunction of Zn^2+^ is linked to a range of CNS disorders, including Alzheimer’s disease, depression and Parkinson’s disease. SLC39A12, a member of the SLC39 zinc transporter family (commonly known as ZIP proteins), promotes neurite outgrowth, neuronal differentiation, and microtubule aggregation and stabilization by transporting zinc to the cytoplasm [31]. Davis et al. have shown that SLC39A12 is crucial for nervous system development [32]. In this study, proteomic analysis revealed an upregulation of SLC39A12, also known as ZIP12, in the hypothalamus of DR rats compared to DIO rats. However, Western blotting did not detect any significant changes in SLC39A12 expression between the two groups. The discrepancy observed might be attributed to the presence of variants or polymorphisms within the SLC39A12 gene. Variations in the length of these gene variants can result in the production of distinct proteins from the same transcript, each potentially exhibiting unique biological functions. Scarr and colleagues identified two such variants of the SLC39A12 protein, designated as NP001138667.1 and NP689938.2 [33], which may contribute to the differential expression and function observed in our study. We will verify the expression of polymorphisms and variants to reveal the potential molecular mechanism in the future. Iron metabolism also plays a vital role in various brain functions. Disruptions in iron homeostasis have been associated with several neurodegenerative diseases. These findings suggest that the abnormal expression of ion-binding proteins might underlie cognitive impairments, learning defects, and diminished neuronal activity in DIO rats.

Compared to DIO rats, DR rats exhibited a significant upregulation of six proteins in the hypothalamus: RHOX12, BASP1, CCDC196, SLC39A12, HSPA4, and RSBN1. Rhox12, a member of the Rhox (reproductive homeobox) gene cluster located on the X chromosome, is primarily expressed in reproductive tissues and the placenta [34]. Although the role of Rhox12 in obesity is yet to be explored, it may be implicated in the enhanced reproductive capacity in rodents. BASP1, a member of the brain acid soluble protein family, is associated with neuron growth and cytoskeletal regulation. It is highly expressed in the nervous system, as well as in the kidney, testicles, spleen, and thymus [35]. Notably, increased expression of BASP1 has been detected in the 61.5% metastatic tumor. Additionally, under stress conditions, BASP1 has been found to regulate progenitor cell polarity and neuronal development. These findings suggest that higher levels of BASP1 could potentially contribute to the development of obesity by modulating neuronal activity. HSPA4, a member of the heat shock protein A family, has been predominantly studied in cancer contexts, including breast, lung, and prostate cancer. In the CNS, HSPA4 has been shown to inhibit the expression of inflammatory cytokines in microglia [36]. In a Parkinson’s mouse model, SIRT1-mediated dehydrated to HSPA4 was shown to reduce neuritis. Elevated HSPA4 expression has been correlated with improved survival in glioma patients [37]. Nevertheless, research examining the relationship between HSPA4 and obesity is limited. In this study, we conducted a comprehensive proteomic analysis of DEPs in the hypothalamus of DIO and DR rats to identify potential targets for improving obesity treatment. However, there are several limitations in this study. Firstly, since our research focuses on rats, further research is required to validate whether these findings are applicable to other rodents, such as mice. Secondly, our analysis of DEPs may have overlooked other potential therapeutic targets. Thirdly, although we have determined that the expression of four proteins is consistent with the group results through Western blotting, the specific role in obesity needs to be further investigated.

In summary, our study identified 31 DEPs in the hypothalamus of DR and DIO rats, providing insights into the molecular mechanisms underlying energy balance and resistance to obesity. Proteins such as TRPV4 and CaMKV emerge as promising targets for future obesity treatment. This work enhances our understanding of the proteomic changes associated with obesity and lays the groundwork for the development of novel therapeutic strategies.

## 4. Materials and Methods

### 4.1. Animals

Male SD rats, aged 8 to 10 weeks, were used for this study. The animals were maintained in a controlled laboratory environment with a temperature range of 21–23 °C, humidity levels between 50% and 60%, and a 12 h light-dark cycle in an air-conditioned facility. All experimental procedures were conducted in accordance with the ethical requirements of the Animal Ethics Committee of Tianjin Medical University.

The rats were acclimatized to laboratory conditions for 1 week before the experiment. Following acclimatization, the animals were fed a chow fat diet (CFD, containing 3.8 kcal/g) or a high-fat diet (HFD, containing 4.77 kcal/g) for 12 weeks. According to the weight gain after 12 weeks and the type of diet fed, we divided the rats into three groups: rats fed the chow diet (control, n = 10); rats fed the HFD that gain more weight than the 1.2 fold weight of control group (diet-induced obesity, DIO, n = 10); rats fed the HFD that gained less weight than the average of the control group (diet-induced obesity resistance, DR, n = 10). As previously reported, the CFD contained 10% fat, 70% carbohydrate, and 20% protein (3.85 kJ, 10% fat calories, Beijing, China); HFD is composed of 45% fat, 35% carbohydrate, and 20% protein (4.73 kJ, 45% calories from fat, Beijing, China) [15]. Food intake was measured daily, and body weight was monitored weekly. Rats in the HFD group whose body weight exceeded 1.2 times the average weight of the control group were classified as obese (DIO). Rats in the HFD group whose body weight was lower than the control group’s average were classified as resistant to obesity (DR). At the end of the experiment, epididymal WAT, BAT, blood samples, and hypothalamic tissues were collected and stored at −80 °C for further analysis.

### 4.2. Body Mass Analysis

Body composition, including fat mass and lean mass, was assessed using X-ray absorptiometry as described by Xi et al. [38].

### 4.3. Hematoxylin and Eosin Staining

WAT and BAT samples were fixed in a 4% polymerized formaldehyde for 24 h. The fixed tissue was dehydrated through a graded ethanol series (75%, 80%, 90%, 95%, and 100%), cleared with xylene, and embedded in paraffin. Tissue sections, 6 μm in thickness, were prepared and stained with hematoxylin-eosin (H&E). Microscopic analysis was conducted at 400× magnification, ensuring that all histological examinations were performed in a blind manner. The average size of the adipocytes was quantified using ImageJ software (Verson 1.8.1).

### 4.4. Blood Lipid Analysis

Serum levels of total cholesterol, triglycerides, HDL-C, and LDL-C were measured using an immunosorbent assay following the manufacturer’s protocols (Beijing, China).

### 4.5. Protein Extraction and Proteomics SAMPLE Preparation

Hypothalamic tissues were subjected to sonication on ice three times using a high-intensity processor in a lysis buffer containing 8 M urea and a 1% protease inhibitor cocktail. The lysate was centrifuged at 12,000× *g* for 10 min at 4 °C to remove cellular debris. The supernatant was transferred into new tubes, and protein concentrations were measured using a BCA assay kit. Equal amounts of protein were precipitated 2–3 times with pre-cooled acetone. After drying the precipitate, 200 mM TEAB (triethylammonium bicarbonate) was added, and the precipitate was sonicated until dissolved. Proteins were digested with trypsin at a 1:50 ratio overnight. The following day, dithiothreitol was added to a final concentration of 5 mM and reduced to 56 °C for 30 min to reduce the proteins. Iodoacetamide was subsequently added and incubated at room temperature in the dark for 15 min.

### 4.6. TMT Labeling

Trypsin-digested peptides were desalted using Strata X C18 (Phenomenex, Torrance, CA, USA) cartridges, freeze-dried, and vacuum-sealed. The peptides were reconstituted in 0.5 M TEAB and labeled using a TMT kit according to the manufacturer’s instructions. Briefly, the labeling reagent was thawed, dissolved in acetonitrile, and incubated with the peptide mixtures at room temperature for 2 h. The labeled peptides were then mixed, desalted, and freeze-dried under vacuum.

### 4.7. HPLC Fractionation

Peptide fragments were fractionated using high-pH reverse-phase HPLC (Agilent 300Ex tend C18 column, 5 μm particles, 4.6 mm inner diameter and 250 mm length, Santa Clara, CA, USA). The peptides were separated using an acetonitrile gradient of 8~32% over 6 min. The resulting fractions were pooled into nine combined fractions and dried using freeze centrifugation.

### 4.8. Liquid Phase Enzyme Analyzer Using LC-MC/MC Analysis

Peptides were dissolved in solvent A (0.1% formic acid and 2% acetonitrile) and separated using an EASY-nLC 1200 ultrahigh-pressure liquid chromatography system. Mobile phase A contained 0.1% formic acid and 2% acetonitrile, while mobile phase B contained 0.1% formic acid and 80% acetonitrile. The gradient elution was set as follows, 6–28% B for 0–42 min, 28–40% B for 42–50 min, and 40–50% B for 50–54 min, and 100% B for 54–60 min, at a flow rate of 300 nL/min.

Peptides were ionized in a nano-spray ion source and analyzed using a Q Exactive HF-X MS (Waltham, MA, USA) mass spectrometer. Data acquisition was conducted using a data-dependent acquisition mode, with automatic gain control set to 1 × 10^5^, a signal threshold of 83,000 ions/s, and a maximum injection time was 60 ms. The dynamic exclusion time for tandem mass spectrum scans was set to 30 s to prevent repeated scans.

### 4.9. Bioinformatics Analysis

GO analysis was conducted to annotate the functions of DEPs in terms of biological processes, cellular components, and molecular functions. Enrichment analysis was performed using the KEGG pathway to identify associated signaling pathways. Protein trimerization and functional predictions were carried out using the Inter-Pro database.

### 4.10. Western Blotting Analysis

Hypothalamic tissue was homogenized in radioimmunoprecipitation assay buffer (Solarbio, Beijing, China) supplemented with phosphatase inhibitor (Roche, Basel, Sweden) and PMSF (Solarbio, China). After incubation for 30 min on ice, lysate was centrifuged at 12,000× *g* for 30 min. Protein concentration was measured using bicinchoninic acid assay (Solarbio, China). Protein samples (30 µg) were separated by 10% SDS-PAGE and transferred onto polyvinylidene fluoride (PVDF) membranes (Millipore, Bedford, MA, USA). Next, the membrane was sealed with 5% skim milk at room temperature to prevent non-specific protein binding. Thereafter, membranes were incubated with specific antibodies against TRPV4 (Abclonal, Wuhan, China, A5660, 1:1000), RSBN1 (bioss, Beijing, China, bs-18865R, 1:1000), BASP1 (Huabio, Hangzhou, China, ER1904-13, 1:1000), SLC39A12 (Huabio, Hangzhou, China, ER1706-33, 1:1000) HSPA4 (proteintech, Wuhan, China, 21206-1-AP, 1:1000), CaMKV (proteintech, Wuhan, China, 14788-1-AP, 1:1000), and GAPDH (Abways, Shanghai, China, P04406, 1:10,000) at 4 °C overnight. After three times washing in TBST for 30 min, the blots were exposed to a secondary antibody (Cell Signaling Techology, Danvers, MA, USA, 7074s, 1:10,000). Then, the blots were again washed three times and signals were visualized by chemiluminescence (Millipore, Bedford, MA, USA). The protein intensities were analyzed using Image J.

### 4.11. Statistical Analysis

Values are presented as mean ± SEM. Data analysis was performed using GraphPad Prism (version 8, GraphPad Software, San Diego, CA, USA). For more than two groups, data were analyzed for statistical significance using one-way ANOVA and LSD. *p* value < 0.05 were considered statistically significant.

## Figures and Tables

**Table 1:** The increased differentially expressed proteins in DR group.

Increased Proteins	Gene Name	Accession	Fold Increase	*p* Value
Reproductive homeobox on X chromosome 12	*Rhox12*	A6JMJ3	3.318	7.8339 × 10^−5^
Lysine-specific demethylase 9	*Rsbn1*	D4A1U7	3.174	0.00012354
Solute carrier family 39-member 12	*Slc39a12*	D4A8R5	2.858	0.00013862
Coiled-coil domain-containing 196	*Ccdc196*	D4A4F0	2.498	0.00018022
Brain acid soluble protein 1	*Basp1*	Q05175	1.618	0.040576
Heat shock 70 kDa protein 4	*Hspa4*	O88600	1.534	0.023158
Mitochondrial ribosomal protein L28	*Mrp128*	D3ZJY1	1.308	0.0061432
Actin-binding LIM protein 2	*Ablim2*	A0A0G2JXC7	1.284	0.048341
Metabotropic glutamate receptor 8	*Grm8*	M0RBY2	1.264	0.048403
Guanine nucleotide-binding protein G	*Gng8*	P63077	1.259	0.048143
Bublin coiled coil protein	*Bbln*	D3ZBT2	1.249	0.039317
Leucine-rich repeat flightless-interacting protein 2	*Lrrfip2*	Q4V7E8	1.238	0.033423
COMM domain containing 8	*Commd8*	B0K015	1.217	0.0074358
Glucan (1,4-alpha-), branching enzyme 1 (Fragment)	*Gbe* *l*	A0A096MJY6	1.213	0.031024

**Table 2:** The decreased differentially expressed proteins in DR group.

Decreased Proteins	Gene Name	Accession	Fold Increase	*p* Value
CaM kinase-like vesicle-associated protein	*Camkv*	A0A0G2K1R5	0.335	0.04622
Transient receptor potential cation channel subfamily V member 4	*Trpv4*	Q9ERZ8	0.421	0.044919
Kirre-like nephrin family adhesion molecule 3	*Kirrel3*	A0A0G2K5L4	0.668	0.0119211
KRIT1, ankyrin repeat-containing	*Krit1*	G3V8Z6	0.71	0.0063187
Endosome-lysosome associated apoptosis and autophagy regulator 1	*Elapor1*	F1MAB2	0.723	0.0057844
Zinc finger MYM-type-containing 3 (Fragment)	*Zmym3*	A0A096MK81	0.742	0.029479
Cathepsin F	*Cts* *f*	Q499S6	0.746	0.0145954
AP complex subunit sigma	*Ap1s1*	B5DFI3	0.746	0.0029365
Protein tyrosine phosphatase, receptor type, G	*Ptprg*	A0A0G2K561	0.751	0.0060037
EKC/KEOPS complex subunit Tprkb	*Tprkb*	Q5PQR8	0.769	0.024203
EBP-like OS = Rattus norvegicus	*Ebpl*	D3ZXC8	0.774	0.0131801
Guanylate cyclase activator 1A	*Guca1a*	D3ZII9	0.775	0.0118963
Selenoprotein P	*Selenop*	P25236	0.78	0.044981
Homeobox protein engrailed-like	*En1*	A0A0G2JT82	0.788	0.040136
Contactin-5	*Cntn5*	F1M173	0.79	0.048221
N-acetyltransferase 8 (GCN5-related) family member 1	*Nat8f1*	V9GZ80	0.806	0.0086754
Leucine-rich repeat transmembrane protein FLRT3	*Flrt3*	B1H234	0.812	0.039741

**Table 3:** List of differentially upregulated proteins from DR vs DIO group (fold changes exceed 1.5 times and *p* value < 0.05).

Protein Accession	Protein Description	Gene Name	MW [kDa]	DR/DIO Ratio	Regulated Type	DR/DIO *p* Value
Q4TU71	Reproductive homeobox on X chromosome 12 OS = Rattus norvegicus OX = 10,116 GN = Rhox12 PE = 2 SV = 1	*Rhox*	20.262	3.318	Up	0.00008
Q05175	Brain acid soluble protein 1 OS = Rattus norvegicus OX = 10,116 GN = Basp1 PE = 1 SV = 2	*Basp1*	21.79	1.618	Up	0.040576
A0A0G2JXV1	Coiled-coil domain-containing 196 OS = Rattus norvegicus OX = 10,116 GN = Ccdc196 PE = 4 SV = 1	*Ccdc196*	19.459	2.498	Up	0.000180219
D4A8R5	Solute carrier family 39 member 12 OS = Rattus norvegicus OX = 10,116 GN = Slc39a12 PE = 4 SV = 3	*Slc39a12*	75.585	2.858	Up	0.00013862
O88600	Heat shock 70 kDa protein 4 OS = Rattus norvegicus OX = 10,116 GN = Hspa4 PE = 1 SV = 1	*Hspa4*	94.055	1.534	Up	0.023158
D4A1U7	Round spermatid basic protein 1 OS = Rattus norvegicus OX = 10,116 GN = Rsbn1 PE = 4 SV = 1	*Rsbn1*	89.286	3.174	Up	0.00012354

## Data Availability

The datasets presented in this study can be found in online repositories.

## Supplementary Materials

The supporting information can be downloaded at: https://www.mdpi.com/article/10.3390/ijms26052296/s1.

## Abbreviations

The following abbreviations are used in this manuscript:

DIO	Diet-induced obesity
DR	Diet-induced obesity resistance
HDL-C	High-density lipoprotein cholesterol
LDL-C	Low-density lipoprotein cholesterol
CFD	Chow-fat diet
HFD	High-fat diet
WAT	White adipose tissue
BAT	Brown adipose tissue
HE	Hematoxylin-Eosin
HPLC	reverse-phase high performance liquid chromatography
GO	Gene ontology
KEGG	Kyoto Encyclopedia of Genes and Genomes
HSPA4	Heat Shock Protein A Family 4
BASP1	Brain acid soluble protein 1
TRPV4	Transient receptor potential cation subfamily V member 4
TMT	Tandem Mass tag
DEPs	differentially expressed proteins
TEAB	triethylammonium bicarbonate
SD	Sprague–Dawley
FDR	false discovery rate
GUCA1A	guanylate cyclase activator 1A
SLC39A12	solute carrier family 39-member 12
ABLIM2	actin-binding LIM protein 2
ZMYM3	Zinc finger MYM-type-containing 3
CNS	central nervous system
PVDF	polyvinylidene fluoride
