# Supplementary figures and images for: Comparative Hypothalamic Proteomic Analysis Between Diet-Induced Obesity and Diet-Resistant Rats
Source: Int J Mol Sci. 2025 Mar 5;26(5):2296. doi: 10.3390/ijms26052296 (PMC11899849; doi:10.3390/ijms26052296)

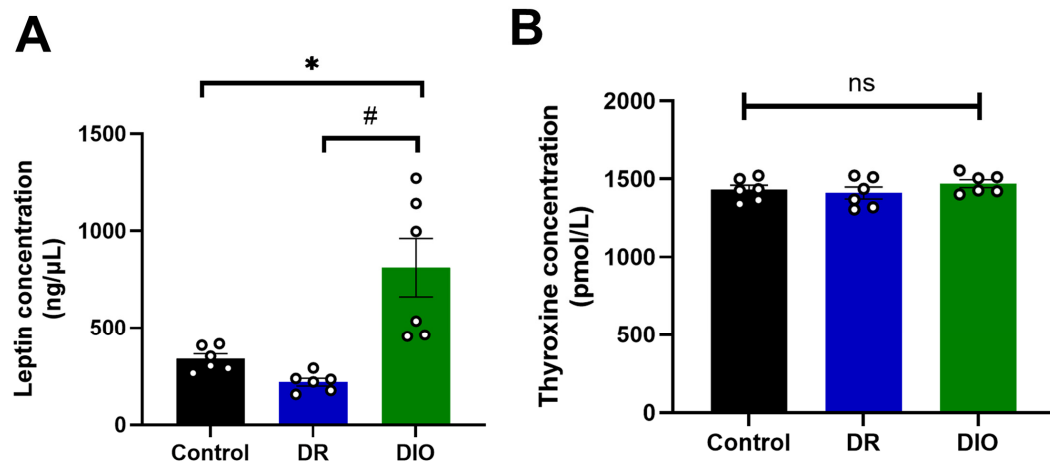

**Figure S1.** The concentrations of leptin and thyroxine hormone in the serum (A-B).

Supplement: Supplementary file 1 [file ijms-26-02296-s001.zip › ijms-3498672-supplementary.pdf]
